# Determination of the Optical GAP in Thin Films of Amorphous Dilithium Phthalocyanine Using the Tauc and Cody Models

**DOI:** 10.3390/molecules170910000

**Published:** 2012-08-24

**Authors:** María Elena Sánchez-Vergara, Juan Carlos Alonso-Huitron, Arturo Rodriguez-Gómez, Jerry N. Reider-Burstin

**Affiliations:** 1 Faculty of Engineering, Anahuac University-Mexico North, Huixquilucan, Estado de México 52786, Mexico; Email: jnreider@anahuac.mx; 2 Institute of Materials Research, National Autonomous University in Mexico, México D.F. 04510, Mexico; Email: alonso@iim.unam.mx (J.C.A.-H.); arturo_r_g@yahoo.com.mx (A.R.-G.)

**Keywords:** thin films, organic semiconductors, optical properties

## Abstract

Semiconducting thin films were grown on quartz substrates and crystalline silicon wafers, using dilithium phthalocyanine and the organic ligands 2,6-dihydroxyanthraquinone and 2,6-diaminoanthraquinone as the starting compounds. The films, thus obtained, were characterized by Fourier Transform infrared (FTIR), fast atomic bombardment (FAB+) mass and ultraviolet-visible (UV-Vis) spectroscopies. The surface morphology of these films was analyzed by means of atomic force microscopy (AFM) and scanning electron microscopy (SEM). It was found that the temperature-dependent electric current in all cases showed a semiconductor behavior with conductivities on the order of 10^−6^·S cm^−1^, whereas the highest value corresponded to the thin film based upon the bidentate amine. The Tauc and Cody optical band gap values of thin films were calculated from the absorption coefficients and were found to be around 1.5 eV, with another strong band between 2.3 and 2.43 eV, arising from non-direct transitions. The curvature in the Tauc plot influencing the determination of the optical gap, the Tauc optical gap corresponding to the thicker film is smaller. The dependence of the Cody optical gap on the film thickness was negligible.

## 1. Introduction

Optical absorption in amorphous semiconductors continues to be the focus of considerable study. In a defect-free crystalline semiconductor, the absorption spectrum abruptly terminates at the energy gap. In contrast, in an amorphous semiconductor, a tail in the absorption spectrum encroaches into the gap region [1]. This tail in the optical absorption spectrum, arising as a consequence of the disorder which characterizes these semiconductors, makes the absorption edge of an amorphous semiconductor difficult to define experimentally. As a result, several models have been developed to describe the electronic transition parameters such as the band gap and the peak transition energy [2]. For many years, the Tauc model has served as the standard empirical model whereby the optical gap of an amorphous semiconductor may be determined. The Tauc model has been applied to amorphous phthalocyanines films, as well. However, few studies have been made to check their validity in the case of two central metal atoms. It is worthwhile noting that these kinds of studies have attracted interest in recent works due to their unique properties, such as semiconductivity and chemical stability [3,4]. The high thermal stability of metallophtalocyanines enables the deposition of thin films through high-vacuum vaporization, as this method does not lead to the breakdown of the synthesized materials. Metallophthalocyanines usually present a given crystalline form in thin films or end up being amorphous, depending mainly upon the molecular self-stacking ability of the derivative, but also on the thin film fabrication procedure [5]. Metallophtalocyanine thin films have been proposed for gas sensing applications [6], solar cells, organic field-effect transistors and OLEDs [7]. These developments have been instrumental in stimulating renewed interest regarding the electrical properties of a wide range of phthalocyanine thin films and in their structural features, as well [6]. The phthalocyanine ligands have rich coordination chemistries and can give rise to complexes with most metal elements [8]. The coordination of phthalocyanine ligands with lithium atoms forms monometallo- or dimetallo-phthalocyanines. The oxidation of dilithium phthalocyanine (Li_2_Pc) generates monolithium phthalo-cyanine (LiPc) which possesses a relatively high electric conductivity and has been recognized as an intrinsic molecular semiconductor [9].

A simple method has been developed to synthesize an amorphous molecular semiconductor from the chemical reaction of dilithium phthalocyanine and bidentate ligands like 2,6-dihydroxyanthraquinone (anthraflavic acid) and 2,6-diaminoanthraquinone (compounds Li_2_PcL1 y Li_2_PcL2, respectively), followed by a thermal evaporation. The film characterization was performed by using SEM and AFM techniques and the electrical conductivity was measured at different temperatures in order to evaluate the conductivity behavior of these films. In addition, the determination of some important optical parameters, related to the principal optical transitions in the UV-Vis region, is reported as well. Application of Tauc and Cody models was made to the deduction of a band gap. The optical gap dependence upon the thickness of these thin films was evaluated. The application of the Tauc and the Cody model enabled us to establish some important correlations between film structure and optical absorption properties. 

## 2. Results and Discussion

**Figure 1:**
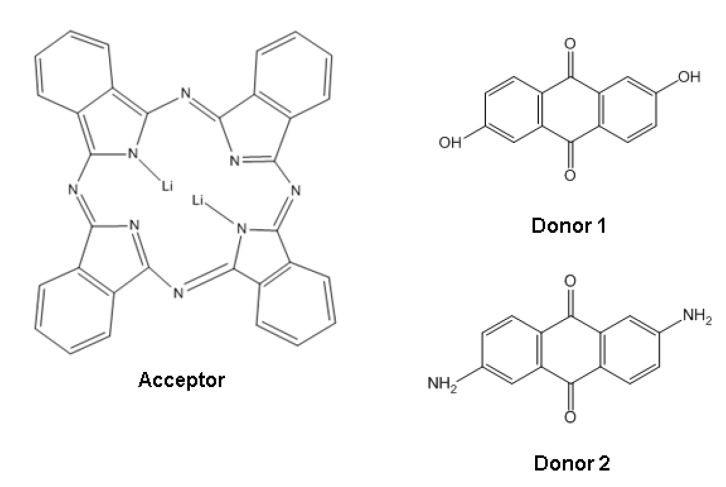
Acceptor and donor schemes.

The electronic acceptor dilithium phthalocyanine was made to react with *n* moles of the organic donor (L1 or L2; see Figure 1). The dilithium phthalocyanine was selected for this study because it is a planar molecule and consists of four isoindole units linked by aza nitrogen atoms and two surrounding lithium atoms. A major property related to this structure is its thermal stability which makes it suitable for sublimation without breaking down [6,10].

**Figure 2:**
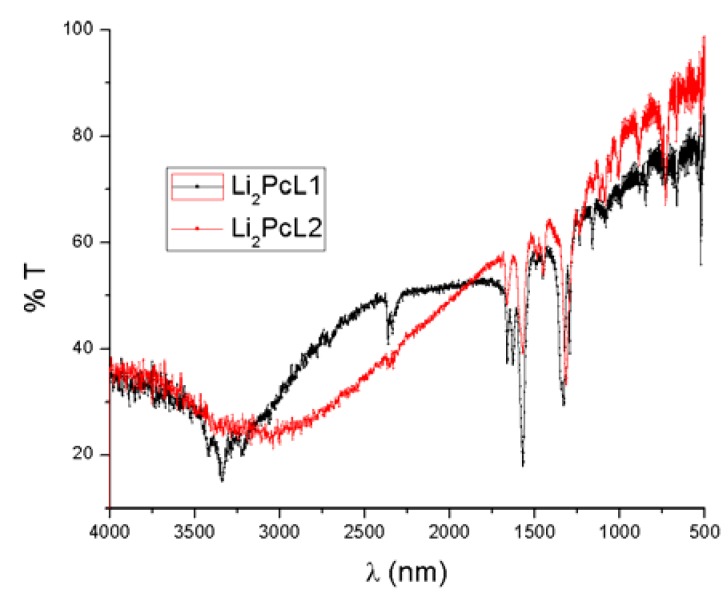
Infrared absorption spectra of Li_2_PcL1 and Li_2_PcL2.

The purpose of the IR spectroscopic studies on these thin films was to identify the most important and representative bonds of the different synthesized compounds. From these studies we were able to determine any significant chemical changes which occurred in these materials during the thermal evaporation deposition procedure. Figure 2 shows infrared absorption spectra of molecular materials in KBr pellet form. Due to the thermal stability of these compounds, chemical changes or reactions are not expected to occur. 

Table 1 shows characteristic bands for these compounds on thin film samples. The absorption peaks lie predominantly in the fingerprint region [11]. The peaks responsible for carbon/nitrogen stretching and bending occur at 1587, 1487, 1328, 1282, and 1057 cm^−1^. The peaks at 1167, 1115 and 779 cm^−1^ result from the interaction of carbon with the peripheral ring hydrogen atoms. The peak at 1093 cm^−1^ results from a carbon/carbon stretch within the macrocyclic ring. Notable peaks also occur at 1707, 881 and 728 cm^−1^, all of them representing other atomic interactions within the macrocyclic ring of Li_2_Pc [11]. Additionally it may be noticed that the material from the thin film Li_2_PcL1 and Li_2_PcL2 exhibits a C=O functional group with wavelengths of 1677 and 1664 cm^−1^, respectively. When compounds are presented in the thin film format, the associated spectrum shows slight changes in the maxima absorption location because, in any thin film structure deposited by thermal evaporation, internal stresses exist which affect the angles and the energies of the intramolecular bonds. Moreover, the IR spectra show the same absorption bands for the thin films as those of the original powders used for evaporation. These results strongly suggest that thermal evaporation is a molecular process which does not change the relative chemical composition of the synthesized compounds. Thus, the deposited films are formed by the same macro-ions as those pertaining to the original synthesized powders.

**Table 1:** IR (cm^−1^) characteristic bands of the synthesized materials for powder and thin film sample formats.

Compound	ν (C–H) (cm^−1^)	ν (C–C) (cm^−1^)	ν (C=N) (cm^−1^)	ν (C=O) (cm^−1^)
Li_2_Pc	1167, 1115, 779	1093	1587, 1487, 1328, 1282, 1057	-
Li_2_PcL1Powder	1156, 1116, 782	1090	1330, 1293, 1068	1677
Li_2_PcL1Thin Film	1157, 1110, 780	1093	1329, 1290, 1065	1674
Li_2_PcL2Powder	1153, 1116, 780	1095	1312, 1279, 1067	1664
Li_2_PcL2Thin Film	1152, 1116, 780	1094	1310, 1282, 1067	1665

Despite the low solubility of the samples, the FAB^+^-mass spectra show fragments with the appropriate isotopic ratio representing the dilithium phthalocyanine moiety, [C_32_H_16_N_8_Li_2_]^+^ (526 *m/z*), and fragments containing the macrocycle and one ligand (Li_2_Pc/ligand), confirming the addition of the ligand to the Li_2_Pc units. In the case of Li_2_PcL1 fragments, containing one Li_2_Pc and one molecule of anthraflavic acid (732 *m/z*), and for Li_2_PcL2 fragments, containing one Li_2_Pc and one amine (744 *m/z*), all the spectra display additional low intensity signals for heavier fragments up to 900 *m/z*. The presence of the characteristic functional groups of the molecules which make up the Li_2_PcL1 and Li_2_PcL2 thin films, obtained through IR spectroscopy, as well as the FAB^+^-mass spectra, help complement the results regarding their stability.

**Figure 3:**
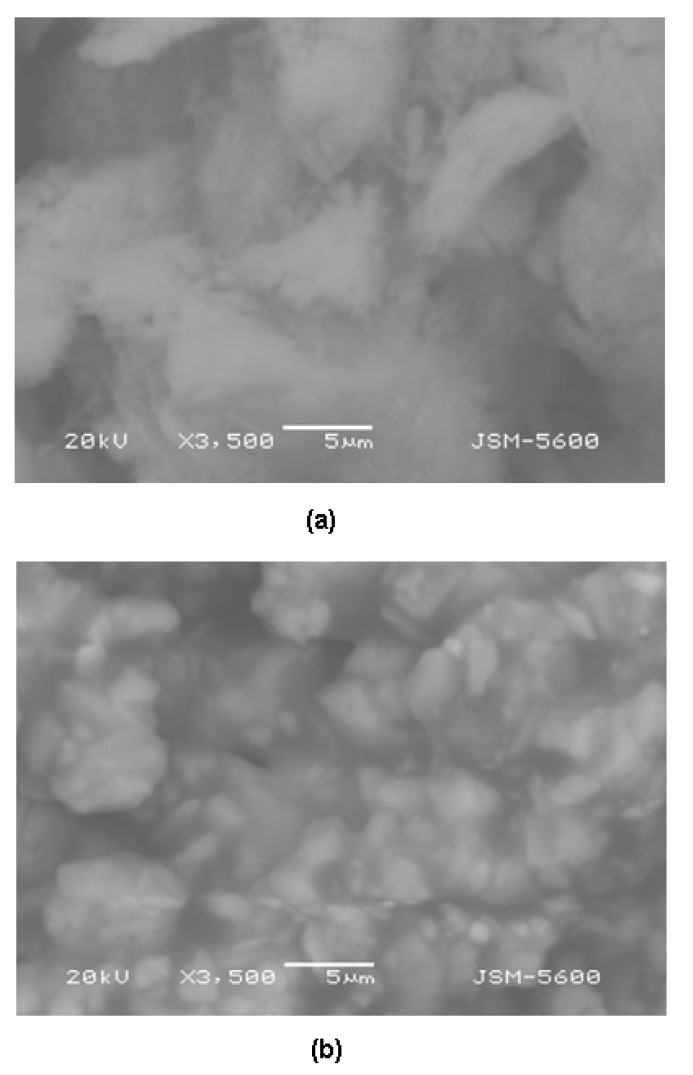
SEM micrographs of (**a**) Li_2_PcL1 and (**b**) Li_2_PcL2 thin films at 3,500×.

The SEM micrographs of the Li_2_PcL1 and Li_2_PcL2 thin films are shown in Figure 3. It may be observed that the surface morphology differs between these two materials. Notwithstanding the effort to maintain a constant deposition parameter, the different melting point for these complexes might prove to be an influence on the resultant morphologies. The thin film synthesized from the anthraflavic acid presents a m.p. of 310 °C, whereas the film synthesized from amine melts at 375 °C. Similar results have been obtained for thin films based upon phthalocyanine and its complexes (magnesium, manganese, iron, cobalt, zinc and lead phthalocyanine) [12,13]. Furthermore, the observed morphology for all materials in the SEM micrographs display an amorphous appearance; this being found in films based upon different materials whenever the low-pressure evaporation technique is employed for their deposition [3,4,5,10]. During the deposition process, when the molecules reach that portion of the substrate at the lowest temperature, their kinetic energy does not suffice in order for them to posses a high-enough surface mobility. Therefore, the long-reach order, characteristic of crystals, is not achieved and an amorphous film structure results.

**Figure 4:**
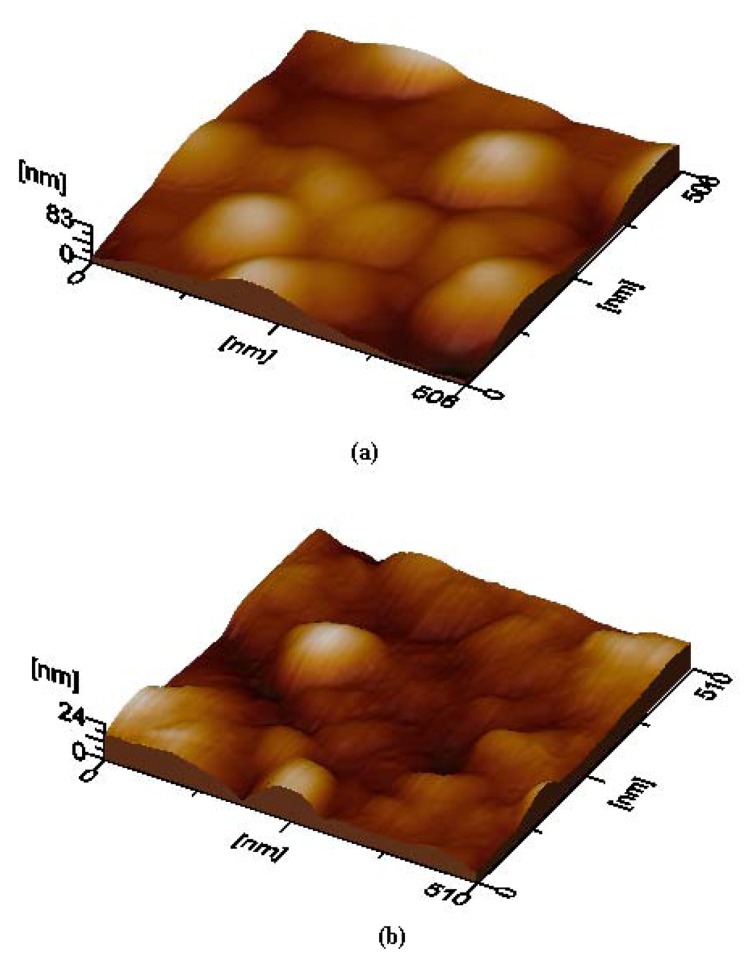
3D-micrographs obtained by AFM, showing the surface morphology of thin films deposited on quartz slices from: (**a**) The Li_2_PcL1 sample and (**b**) sample, Li_2_PcL2 respectively.

AFM is used to evaluate the surface quality of the deposited films. Figure 4 shows 3D-micrographs obtained from the Li_2_PcL1 and Li_2_PcL2 thin films. In a general sense, the agglomeration of small particles in order to generate huge rounded grains with a reasonably heterogeneous distribution at large micrometric length scales may be appreciated. The root mean square (rms) roughness, as evaluated from the AFM measurements of the thin films, is 14.3 nm for Li_2_PcL1 and 4.3 nm for Li_2_PcL2. The difference in the roughness values may be related to the different bidentate ligand in each molecular material. It appears that the amine group in Li_2_PcL2 acts as a Lewis base with a pair of available electrons. The nitrogen atom intervenes in the delocalized orbital, the aromatic ring being part of it, delocalizing the non-shared amine group electron pair within this structure. Hence, the amine group activates the aromatic ring into an oxidation state, due to the influence of electrophilic agents like oxygen in air, as well as other oxidation products, which might affect the film’s surface. 

In order to investigate the electrical behavior of the synthesized materials, the variation of electrical conductivity as a function of temperature in thin films was evaluated. The measurement took place over a line on the material that had equal spaces between the test points. Care was taken to ensure that the current involved was low enough to prevent sample heating, together with a high input impedance voltmeter. Electrical measurements point towards a semiconductor characteristic for the two synthesized materials. From the above results, the electrical conductivity of all thin films was evaluated at 298 K (Table 2). Such σ values lie within the semiconductor region (10^−6^ to 10^1^ S cm^−1^) [14]. The compound Li_2_PcL2 exhibits a higher electrical conductivity at room temperature. The electric conductivity values for these films suggest that the addition of the 2,6-diaminoantraquinone favors the charge transport mechanism. It is possible that the conductive anisotropy arises from a columnar disposition of the phthalocyanine-ligand arrangement where the charge transport takes place; the conduction mechanism being mainly based upon the bidentate ligand. Apparently, the electron pair in each amine nitrogen atom becomes a donor group owing to a resonance mechanism. This, in turn, activates the molecule and generates a significant electron delocalization giving rise to a preferential direction for the electric charge transport [3,10].

**Table 2:** Characteristic parameters of thin-films under investigation ^a^.

Sample	*d_1_* (Å) ^b^	*Tauc Eg* (eV) ^c^	*Cody Eg* (eV) ^d^	*d_2_* (Å) ^b^	*Tauc Eg* (eV) ^c^	*Cody Eg* (eV) ^d^	*d_3_* (Å) ^b^	*Tauc Eg* (eV) ^c^	*Cody Eg* (eV) ^d^	σ_298 K_(Scm^−1^) ^e^
**Li_2_PcL1**	4049	1.50, 2.30	1.50, 2.31	2893	1.54, 2.40	1.50, 2.31	1736	1.55, 2.43	1.54, 2.34	2.0 × 10^−5^
**Li_2_PcL2**	6447	1.49, 2.30	1.47, 2.10	4975	1.52, 2.39	1.49, 2.12	2031	1.55, 2.41	1.49, 2.12	4.2 × 10^−5^

^a^ *d*: film thickness; *Eg*: band gap; σ: electrical conductivity; ^b^ ±5% tolerance; ^c^ ±15% tolerance; ^d^ ±15% tolerance; ^e^ ±10% tolerance.

Hopping is a conduction process observed in Li_2_PcL1 and Li_2_PcL2 thin films, similar to the conduction process in thin films of (FePc)K and triclinic PbPc [6]. This general process is known for amorphous materials [6,15,16], for which the lack of long-range order gives rise to a phenomenon known as localization in which the energy levels do not merge into one another, particularly in the region at the edges of the energy bands. The effect of this is that, in order for carriers to be transported through the material and to contribute to the conductivity, they have to proceed by a series of “hops” from one localized energy level to another. Hopping occurs between the various localized energy levels when thermal energy is available. According to Mott and Davis [16], in this type of material the conductivity σ exhibits different behavior in different regions of its log σ *vs.* 1/T characteristic. At higher temperatures thermal excitation of carriers to the band edges is possible and extended-state conductivity can take place, while at lower temperatures, where less thermal energy is available hopping may occur [6].

**Figure 5:**
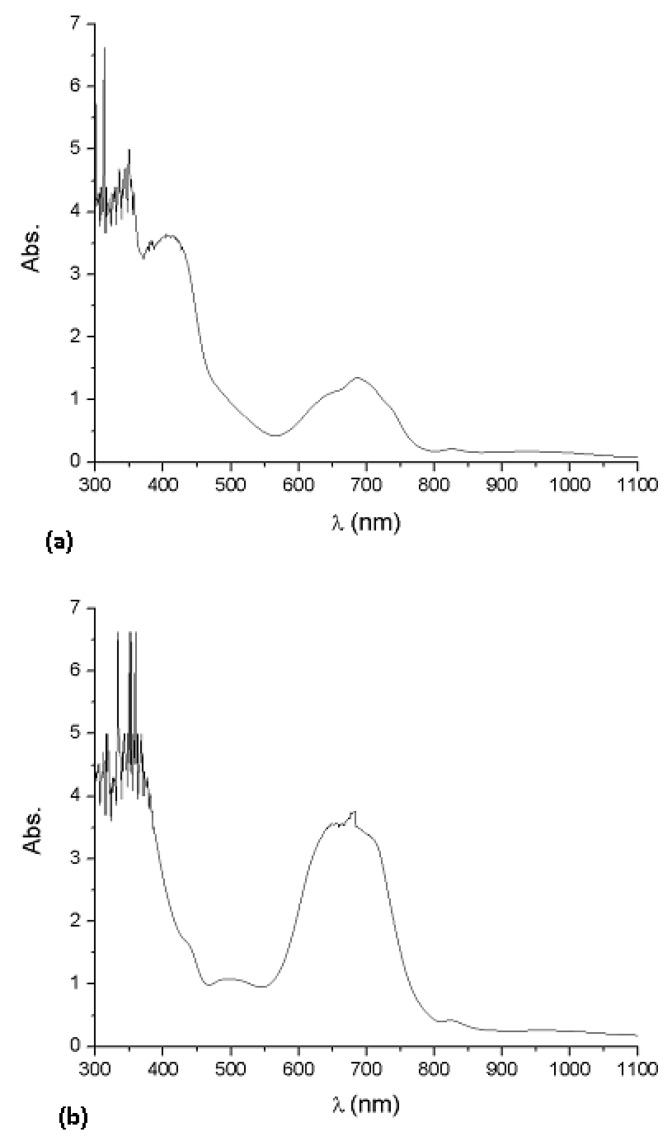
Absorption spectra of the (**a**) Li_2_PcL1 and (**b**) Li_2_PcL2 thin films for 4049 Å and 6447 Å thicknesses, respectively.

Optical absorption measurements are widely used to characterize the electronic properties of materials, through the determination of parameters describing the electronic transitions such as: band gap, valence band tails and excited state lifetime [2]. The band gap is deduced from the ultraviolet/visible absorption spectrum of the thin films. The spectra of Li_2_PcL1 and Li_2_PcL2 contain intense bands in the UV and visible ranges. The absorption spectra of the deposited Li_2_PcL1 and Li_2_PcL2 thin films with thicknesses of 4049 Å and 6447 Å, respectively, are shown in Figure 5. An increase of intensity of the absorption peaks, as the film thickness increases, is found. The UV-Vis spectrum observed for phthalocyanines thin films, originates from molecular orbitals within the aromatic 18π electron system and from overlapping orbitals on the central metal atoms [13]. Li_2_PcL1 and Li_2_PcL2 thin films show a strong Vis band (Q band) around 656 nm and two broad near-UV bands (B or Soret bands) at 370 and 328 nm respectively, consistent with a typical closed-shell metallo phthalocyanine [8,17]. In a similar fashion to the MgPc thin films [7], since the central Li metal atoms are devoid of *d* electrons, the mixing between metal and ligand orbitals is negligible, so that electronic transitions take place between ligand-centered orbitals, all transitions being π–π* in nature. The bands around 370–328 nm are due to electronic transitions between molecules, having an intermediate ionic degree, which conform the synthesized molecular materials. The Q band, can be attributed to the allowed highest occupied molecular orbital (HOMO)-lowest unoccupied molecular orbital (LUMO) (π–π*) transition in the phthalocyanine ring [18]. The maximum absorption peak in the Q band is assigned to a remote wavelength region owing to the coordination of ligand to metallic ions in the phthalocyanines. The presence of this absorption band may be interpreted as an overlap of π orbitals through the ligand. 

**Figure 6:**
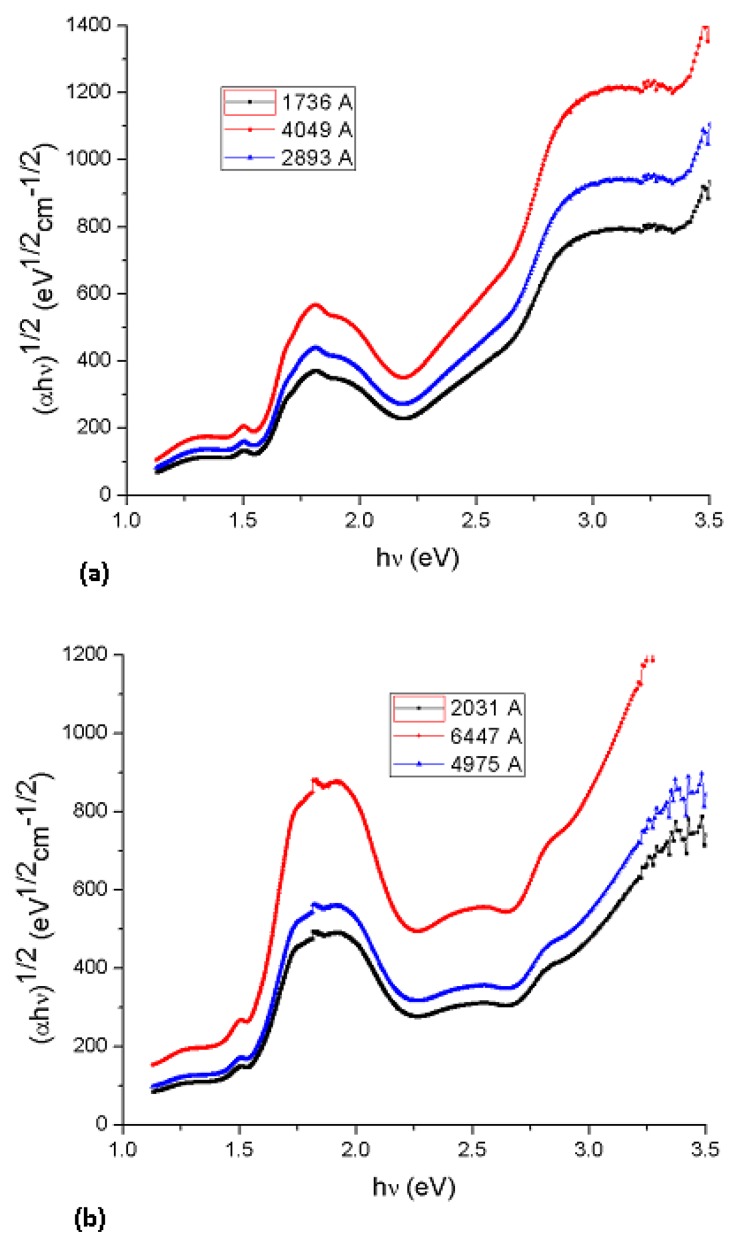
Plot of (α*hν*)^1/2^ *vs*. photon energy *hν* of (**a**) Li_2_PcL1 and (**b**) Li_2_PcL2 thin films.

Several models are used to determine the optical properties of amorphous semiconductors. The most widespread is the Tauc model [2] which allows us to derive the band gap energy E_g_ from E(ε)^1/2^ as function of the incident energy E. The Tauc optical gap associated with the thin films is determined through an extrapolation of the linear trend observed in the spectral dependence of (αhν)^1/2^ over a limited range of photon energies hν [19]: the Tauc optical gap is defined as occurring at the intercept of this linear extrapolation with the abscissa axis [1]. The absorption coefficient *α* near the band edge in many amorphous semiconductors shows an exponential dependence upon photon energy usually obeying the empirical relation [20]:


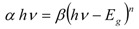
(1)

where β^−1^ is the band edge parameter and *n* is a number characterizing the transition process which may take values 1/2, 1, 3/2 or 2, depending upon the nature of the electronic transitions responsible for the absorption [21]. At this point it may be recalled that, in amorphous semiconductors, optical transitions are described to a first approximation by non-direct transitions with no conservation of electronic momentum, for allowed indirect transitions *n =* *2* [22]. In order to gain some appreciation regarding how the film thickness associated with Li_2_PcL1 and Li_2_PcL2 influences the determination of the corresponding Tauc optical gap, consider Figure 6, in which Tauc optical gap determination are shown for three different film thicknesses for Li_2_PcL1 (4,049, 2,893 and 1,736 Å) and three film thicknesses for Li_2_PcL2 (6,447, 4,975 and 2,031 Å) [19]. Thus, the optical gaps for both indirect transitions could be determined by the extrapolation to zero of the linear regions in the (*αhυ)^½^* = *f(hν)* plots. Figure 6 indicates that Li_2_PcL1 and Li_2_PcL2 thin films have a strong absorption band around 1.5 eV and another strong band between 2.3 and 2.43 eV. Similar results were obtained for NiPc thin films [13], the analysis of the spectral behavior revealing two indirect allowed transitions. Given the nature of the curvature, the Tauc optical gap corresponding to the thicker film is smaller than that of its thinner counterpart (see Table 2). This increase in the Tauc optical gap associated with Li_2_PcL1 and Li_2_PcL2, corresponding to decreasing film thickness, has been attributed to a number of factors [19]. 

**Figure 7:**
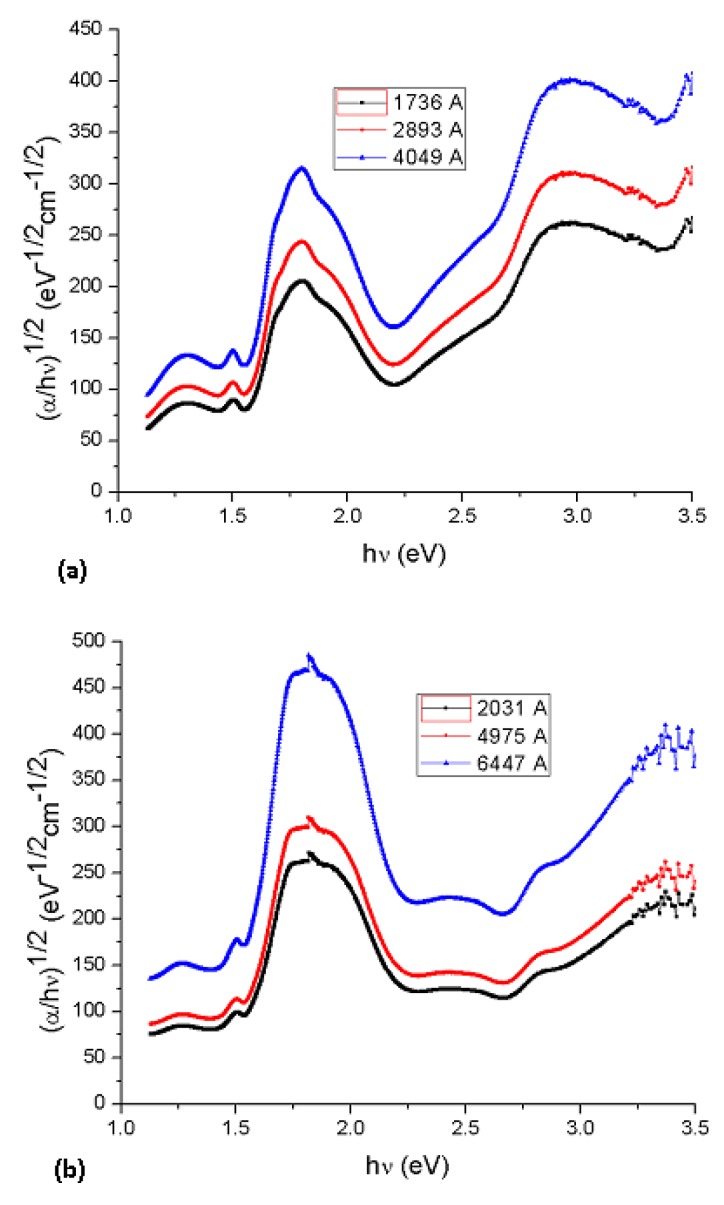
Plot of (α/*h*ν)^1/2^ *vs*. photon energy *hν* of (**a**) Li_2_PcL1 and (**b**) Li_2_PcL2 thin films.

Cody *et al.*, hypothesize that this behavior occurs due to a fundamental curvature in the spectral dependence of (*αhυ)^½^* = *f(hν)* which they hold responsible for the increases in the Tauc optical gap as associated to decreasing film thickness [19]. In accordance to the model, as given by Cody *et al.*, the optical gap associated with Li_2_PcL1 and Li_2_PcL2 thin films should rather be determined by extrapolating the linear trend observed in the spectral dependence of (α/*hν*)^1/2^, over a limited range of photon energies. The abscissa axis intercept of this linear extrapolation, corresponding to the Cody optical gap (Figure 7) on the thickness of the film, became negligible. Cody plots exhibit a much milder curvature than their Tauc counterparts and much milder dependence in the gap associated with Li_2_PcL1 and Li_2_PcL2 thin films thickness than for the case of the Tauc optical gap [19]. Tauc assumes that the momentum matrix element is independent of the photon energy, Cody suggests, instead, that the dipole matrix element is actually independent of the photon energy. Table 2 shows optical gap determinations for Cody model. Even though differences in terms of the activation energy may be perceived in the graphs, similar values may be observed by comparing the results arising from the Tauc and the Cody models. If the measurement error range is taken into account, it may be concluded that these differences are really not important so that any of the two models may be used for calculating the activation energy of organic semiconductors.

In amorphous semiconductors, the optical transitions are dominated, to a first approximation, by the so-called indirect transitions. In these electronic transitions from states in the valence band to states in the conduction band, there is no conservation of the electronic momentum [23]. An alternative explanation may be reached considering the generation of Frenkel-type, tightly-bound excitons [24]. It has been observed [23] that significant charge localization in organic molecular materials leads to a significant difference between the size of the optical gap and the size of the transport gap, which corresponds to the formation energy of a separated free electron and a hole. Whereas the optical gap can be measured by optical absorption spectroscopy, the transport gap can be measured by ultraviolet or inverse photoemission spectroscopy, the latter one being larger than the former by a quantity equal to the binding energy of the Frenkel exciton.

## 3. Experimental

### 3.1. Starting Materials and Chemicals

All chemicals were reagent-grade from commercial suppliers. In this case, 2,6-diaminoanthraquinone and 2,6-dihydroxyanthraquinone anthraquinone (anthraflavic acid) were used in order to generate a double substitution between the organic compound and the dilithium phthalocyanine. Vibrational spectra (4000–400 cm^−1^) were acquired with a Perkin-Elmer model Tensor 27 IR spectrophotometer. The materials intended for the samples were hand-ground in an agate mortar and later pre-mixed with KBr (spec pure). The resulting substances were then pressed into clear pellets. Mass spectra (FAB+) were measured on a 3-nitrobenzyl alcohol support in the positive ion mode on a Jeol JMS-SX102A instrument (Mexico City, México).

Synthesis of Li_2_PcL1: anthraflavic acid (0.12 g, 0.50 mmol) is dissolved in methanol (20 mL) for two hours. Then dilithium phthalocyanine (0.12 g, 0.23 mmol) is added and the mixture refluxed for 72 h, then cooled to room temperature, filtered and washed with methanol. The resulting blue powder was dried under high vacuum, to give a 73% yield of product, which was recrystallized from a 1:1 methanol–water mixture. MS (FAB^+^, DMSO/EtOH) *m/z*: 526 [C_32_H_16_N_8_Li_2_]^+^, 700 [C_46_H_22_N_8_Li_2_]^+^, 732 [C_46_H_22_N_8_O_2_Li_2_]^+^.

Synthesis of Li_2_PcL2: following a similar procedure as for Li_2_PcL1, but using 2,6-diaminoanthraquinone (0.12 g, 0.50 mmol) and dilithium phthalocyanine (0.13 g, 0.25 mmol) a 75% yield of prodyt was obtained, which was recrystallized from a 1:1 methanol-water mixture. MS (FAB^+^, DMSO/EtOH) *m/z*: 526 [C_32_H_16_N_8_Li_2_]^+^, 712 [C_46_H_34_N_8_Li_2_]^+^, 744 [C_46_H_34_N_8_O_2_Li_2_]^+^.

### 3.2. Film Deposition

In order to achieve a high purity for these thin films, a vacuum chamber was used with a diffusion pump and a special molybdenum crucible with a double-grid cover. Quartz fiber was added inside the crucible to avoid the ejection of grains towards the substrate at a temperature of 553 K. The pure material was deposited over quartz and (100) single-crystalline silicon (c-Si), 200 Ω-cm wafers. The quartz substrates were ultrasonically degreased in warm methanol and dried under a nitrogen atmosphere. The substrates underwent chemical etching with a p solution (10 mL HF, 15 mL HNO_3_ and 300 mL H_2_O) in order to remove the native oxide from the c-Si surface. The substrates were kept at 298 K. The evaporation temperature in the boat was 553 K, which is lower than the decomposition temperature of the materials synthesized for this study. This value was measured by means of a chromel-alumel K-type thermocouple. All samples were obtained using the same deposition system, with the crucible and substrates arranged in the same geometry. The base pressure in the deposition chamber before thin-film deposition and the amount of matter inside the crucible were the same in all cases. In spite of these similarities, significant differences in the thickness of the deposited films were detected. According to the data in Table 1, the thin film synthesized from anthraflavic acid and dilithium phtalocyanine ended up being thicker as compared to the film prepared with the 2,6-diaminoanthraquinone bidentate ligand [10]. The substitution of the OH group for NH_2_ in the ligand causes a higher evaporation temperature for this compound.

### 3.3. Thin Film Characterization

Fourier Transform infrared spectra (FTIR) for thin films were recorded with a Nicolet 5-Mx FT-IR spectrophotometer with a resolution of 4 cm^−1^. The surface characterization was made by scanning electron microscopy (SEM) in a Leica Cambridge, Stereoscan 440 model (Mexico City, México). A focal distance of 25 mm and a 20 kV potential were used for all the samples. The surface morphology of films was analyzed by Atomic Force Microscopy (AFM) with a Jeol JSPM-4210 in contact mode with tapping and non-contact silicon cantilevers (NSCIS). The thickness of films was determined by ellipsometry using a Gaetner L117 variable-angle manual ellipsometer with a helium-neon laser as a light source operating at 630 nm. The incidence angle was 70°. The infrared and ellipsometric measurements were carried out in the film deposited onto c-Si substrates. Ultraviolet-visible (UV-Vis) transmission and absorption spectra for films deposited onto naked quartz substrates were obtained with a Shimadzu 260 double-beam spectrophotometer. Electric current as a function of temperature was measured with an applied voltage of 100 V in the ohmic regime, using a Keithley model 230 programmable voltage source and a Keithley model 485 peak-ammeter coupled to an HP3421 data collector.

## 4. Conclusions

New semiconductor materials (Li_2_PcL1 and Li_2_PcL2) were synthesized from dilithium phthalocyanine, 2,6-dihydroxianthraquinone and 2,6-diaminoanthraquinone and deposited by vacuum thermal evaporation. Although the deposited material is amorphous in nature, it is formed by the same chemical unit as that present in synthesized powder. From the electric current values, it is clear that all the molecular thin films show a semiconductor-like behavior after evaporation, with conductivities in the order of 10^−6^–10^−1^ S·cm^−1^ at room temperature. The UV-Vis optical transmission spectra were analyzed in the edge strong absorption region. Li_2_PcL1 and Li_2_PcL2 presented two absorption bands, namely the Q band in the visible region (around 650 nm) and two broad near-UV bands (B bands) at 370 and 328 nm. Using the Tauc and Cody models, the optical gaps associated with thin films was determined. The thickness in the thin films plays an important role in determining the Tauc optical gap; the gap is larger for smaller film thickness. Although the Cody optical gap is less dependent upon film thickness, both models estimate similar results regarding the activation energy. Finally, the value of the calculated optical gaps, the order of magnitude of the electrical conductivities and the possibility to prepare thin films from these materials suggest that it may be possible to apply them for preparing electronic devices.
